# Comparative Plastid Genomics of Green-Colored Dinoflagellates Unveils Parallel Genome Compaction and RNA Editing

**DOI:** 10.3389/fpls.2022.918543

**Published:** 2022-07-11

**Authors:** Eriko Matsuo, Kounosuke Morita, Takuro Nakayama, Euki Yazaki, Chihiro Sarai, Kazuya Takahashi, Mitsunori Iwataki, Yuji Inagaki

**Affiliations:** ^1^Graduate School of Life and Environmental Sciences, University of Tsukuba, Tsukuba, Japan; ^2^Center for Computational Sciences, University of Tsukuba, Tsukuba, Japan; ^3^RIKEN iTHEMS, Saitama, Japan; ^4^Graduate School of Science and Engineering, Yamagata University, Yamagata, Japan; ^5^Asian Natural Environmental Science Center, The University of Tokyo, Tokyo, Japan

**Keywords:** serial secondary endosymbiosis, peDinoflagellates, Pedinophyceae, *Lepidodinium*, RNA editing, complex plastids, plastid replacements

## Abstract

Dinoflagellates possess plastids that are diverse in both pigmentation and evolutionary background. One of the plastid types found in dinoflagellates is pigmented with chlorophylls *a* and *b* (Chl *a* + *b*) and originated from the endosymbionts belonging to a small group of green algae, Pedinophyceae. The Chl *a* + *b*-containing plastids have been found in three distantly related dinoflagellates *Lepidodinium* spp., strain MGD, and strain TGD, and were proposed to be derived from separate partnerships between a dinoflagellate (host) and a pedinophycean green alga (endosymbiont). Prior to this study, a plastid genome sequence was only available for *L. chlorophorum*, which was reported to bear the features that were not found in that of the pedinophycean green alga *Pedinomonas minor*, a putative close relative of the endosymbiont that gave rise to the current Chl *a* + *b*-containing plastid. In this study, we sequenced the plastid genomes of strains MGD and TGD to compare with those of *L. chlorophorum* as well as pedinophycean green algae. The mapping of the RNA-seq reads on the corresponding plastid genome identified RNA editing on plastid gene transcripts in the three dinoflagellates. Further, the comparative plastid genomics revealed that the plastid genomes of the three dinoflagellates achieved several features, which are not found in or much less obvious than the pedinophycean plastid genomes determined to date, in parallel.

## Introduction

Dinoflagellates are a large group of eukaryotic algae, and one of the major primary producers in the aquatic environment. Some species are infamous for causing red tides and producing deadly toxins causing shellfish poisoning (Jeffrey et al., 1975; Carty and Parrow, 2015). The vast majority of the extant dinoflagellates possess (or used to possess) the plastids containing chlorophylls *a* and *c* (Chl *a* + *c*) plus peridinin, the carotenoid uniquely found in this algal group (“peridinin plastids”; dinoflagellates bearing peridinin plastids are termed simply as “peridinin dinoflagellates”; Jeffrey et al., 1975; Zapata et al., 2012). Peridinin plastids were one of the “secondary plastids” derived from red algal endosymbionts and are believed to be established prior to the divergence of the extant dinoflagellates (Archibald, 2009; Keeling, 2010; Sibbald and Archibald, 2020). The plastid genomes in diverse peridinin dinoflagellates comprise multiple mini-circles (Zhang et al., 1999; Barbrook et al., 2019), each of which carries a single or a few genes. Further, the transcripts from mini-circles have been known to undergo post-transcriptional base conversion (base conversion RNA editing), which converts the four nucleotides in transcripts to almost all possible others (Zauner et al., 2004; Dang and Green, 2009; Mungpakdee et al., 2014; Klinger et al., 2018). Such promiscuous base conversion editing in peridinin plastids is distinctive from the RNA editing in organelles of other eukaryotes intensively studied so far [e.g., land plants; Shikanai (2006) and Hao et al. (2021)], leaving the details of the RNA editing machinery in peridinin plastids uncertain.

**FIGURE 1:**
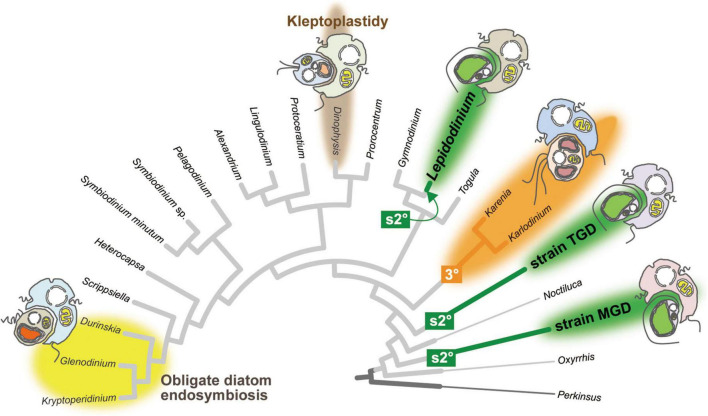
Plastid evolution in dinoflagellates. The cladogram of 22 dinoflagellates and *Perkinsus marina* was prepared by modifying the phylogenetic tree inferred from a 75-protein alignment analyzed in Sarai et al. (2020). The branches of *Lepidodinium*, strain TGD, and strain MGD are colored in green, as their non-canonical plastids were yielded independently from pedinophycean endosymbionts (serial secondary endosymbiosis; labeled as “s2°”). The clade of two kareniacean dinoflagellates, *Karenia* and *Karlodinium*, are colored in orange, as they possess the non-canonical plastids deduced from the endosymbiotic haptophyte (tertiary endosymbiosis; labeled as “3°”). The clade of the dinoflagellates bearing obligate diatom endosymbionts are highlighted by a yellow background. The *Dinophysis* branch is highlighted by a brown background, as some members of the order Dinophysiales retain and utilize the plastids of prey algae temporarily (kleptoplastidy). Non-photosynthetic species are shown by thin branches.

Dinoflagellates have been anticipated to provide clues to understand the evolutionary process transforming an endosymbiotic alga into the host-governed organelle (i.e., plastid), as peridinin plastids have been replaced by phylogenetically diverse algae taken up as the endosymbionts on multiple branches in the tree of dinoflagellates. Figure 1 schematically summarizes the lineages/species bearing “non-canonical plastids” lacking peridinin (Archibald, 2009; Keeling, 2010; Sibbald and Archibald, 2020) and obligate diatom endosymbionts (Takano et al., 2008; Yamada et al., 2017). Members of the family Kareniaceae possess the non-canonical plastids containing Chl *a* + *c* plus 19′-hexanoyloxyfucoxanthin instead of peridinin (Bjørnland et al., 2003; Zapata et al., 2012). Consistent with the pigment composition, phylogenies of plastid genes suggested that the non-canonical plastids in kareniacean dinoflagellates are the product of “tertiary endosymbiosis,” in which an endosymbiotic haptophyte was reduced and integrated genetically into the dinoflagellate host as the plastid (Tengs et al., 2000; Nosenko et al., 2006; Burki et al., 2014; Bentlage et al., 2016; highlighted as “3°” in Figure 1). The plastid gene transcripts of kareniacean dinoflagellates were shown to receive promiscuous base conversion editing, which is similar to but more intense than that of peridinin plastids (Klinger et al., 2018).

Another type of non-canonical plastid, which contains chlorophylls *a* and *b* (Chl *a* + *b*), was found initially in members of the genus *Lepidodinium* (Watanabe et al., 1987, 1990). A phylogenetic study based on multiple plastid genes pinpointed the origin of the *Lepidodinium* plastid as a pedinophycean green alga (Kamikawa et al., 2015). The host phylogeny inferred from the nucleus-encoded ribosomal RNA (rRNA) sequences put *Lepidodinium* spp. within peridinin dinoflagellates, such as *Gymnodinium catenatum* (Saldarriaga et al., 2001; Shalchian-Tabrizi et al., 2006; Matsumoto et al., 2012), suggesting that the ancestral *Lepidodinium* cell replaced the peridinin plastid with a Chl *a* + *b*-containing plastid through the endosymbiotic partnership with a pedinophycean green alga (termed as “serial secondary endosymbiosis;” highlighted as “s2°” in Figure 1). Similar to the non-canonical plastids in kareniacean dinoflagellates, the evidence for the genetic integration of the pedinophycean-derived plastid into the dinoflagellate host has been accumulated (Minge et al., 2010; Matsuo and Inagaki, 2018). More recently, some of us reported the second and third dinoflagellates bearing Chl *a* + *b*-containing plastids, strains MGD and TGD (Nakayama et al., 2020; Sarai et al., 2020). In the host phylogeny inferred from an alignment of 75 nucleus-encoded proteins, *L. chlorophorum*, strain MGD, and strain TGD were distantly related to each other, suggesting that serial secondary endosymbiosis occurred on the three separate branches in the tree of dinoflagellates (the three branches labeled with “s2°” in Figure 1). The plastid phylogeny inferred from plastid small subunit rRNA sequences recovered the groupings described below with high statistical support, namely (i) the monophyly of the three dinoflagellates bearing Chl *a* + *b*-containing plastids and (ii) the sister relationship between the dinoflagellate clade and the clade of *Pedinomonas minor* and *P. tuberculata* (Sarai et al., 2020). Thus, the endosymbiont algae, which gave rise to the Chl *a* + *b*-containing plastids in *L. chlorophorum*, strain MGD, and strain TGD, belong to or are closely related to the genus *Pedinomonas*. Here, we designate the three dinoflagellates bearing pedinophycean-derived plastids as “peDinoflagellates,” and their plastids as “peDinoflagellate plastids.”

We have been interested in extracting the key aspects that enabled serial secondary endosymbiosis by comparative studies of the three peDinoflagellates (Nakayama et al., 2020; Sarai et al., 2020). In the line of our research interest described above, we here evaluate how the plastid genomes of the pedinophycean endosymbionts were modified during serial secondary endosymbioses by comparative plastid genomics. Kamikawa et al. (2015) sequenced the plastid genome of *L. chlorophorum* completely and reported that the peDinoflagellate plastid genome is more compact than that of *P. minor* in terms of the repertory of functionally assignable open reading frames (ORFs). In addition, the *L. chlorophorum* plastid genome has the features that were not found in the pedinophycean plastid genomes; (i) absence of inverted repeats (IRs), (ii) frequent ORF/gene overlapping/fusion, (iii) a deviant genetic code in which AUA codon is assigned as methionine (Met) instead of isoleucine (Ile), and (iv) pseudogenization (Kamikawa et al., 2015). Thus, it is intriguing whether the above-mentioned features found in the *L. chlorophorum* plastid genome are shared with either or both MGD and TGD plastid genomes.

In this study, we sequenced the plastid genome of strain TGD completely and that of strain MGD nearly completely (the latter genome could not be completed due to repeat sequences). The TGD plastid genome is a circular molecule of approximately 71 Kb, while strain MGD possesses the circular plastid genome of approximately 102 Kb. The current study revealed that the three peDinoflagellate plastid genomes shared many of the features that were identified by the comparison between the plastid genomes of *L. chlorophorum* and *P. minor*. In addition, we found RNA editing on plastid gene transcripts in the three peDinoflagellates. Base conversion editing on the plastid gene transcripts appeared to be common among the three peDinoflagellate plastids, while we identified a single case of base insertion editing on the *psaA* transcript in strain TGD. Overall, the patterns of base conversion editing were similar among the dinoflagellates bearing peridinin plastids and the two types of non-canonical plastids except for that of strain MGD. If *L. chlorophorum*, strain MGD, and strain TGD truly established their current plastids separately, the modifications of plastid genomes in the pedinophycean endosymbionts occurred in a highly parallel manner during separate serial secondary endosymbioses.

## Materials and Methods

### DNA Extraction From the Cultured Cells

PeDinoflagellate strains TGD and MGD established in Sarai et al. (2020) have been maintained in our laboratories and were used in this study. The culture conditions of the two strains were the same as described in Sarai et al. (2020). The algal cultures were observed by light microscopy regularly and the cells in confluent cultures were harvested by centrifugation at 1,720 *g* for 10 min. The genomic DNA was extracted from the harvested cells by the cetyl trimethyl ammonium bromide (CTAB) method. The cell pellet was dissolved in 500 μL of CTAB extraction buffer [per 100 mL, 2 *g* CTAB, 10 mL of 1 M Tris–HCl (pH 8.0), 4 mL of 0.5 M ethylenediaminetetraacetic acid (pH 8.0), and 35 mL of 4 M NaCl] at 65^°^C for 1 h. After the cells were completely dissolved, 500 μL of Chloroform:Isoamyl Alcohol Solution [480 μL chloroform: 20 μL Isoamyl Alcohol] was added to the tube. After vigorous vortexing, the aqueous solution was saved in a fresh tube by centrifugation at 13,200 rpm for 5 min at 20^°^C. Isopropanol precipitation was carried out by adding 500 μL of isopropanol to the aqueous solution, followed by centrifugation at 13,200 rpm for 20 min at 4^°^C. The pellet was rinsed with 1 mL of 70% ethanol, and the supernatant was discarded after centrifugation at 13,200 rpm for 5 min at 4^°^C. The DNA pellet was briefly dried up and then dissolved into 50 μL of sterile distilled water.

### Sequencing, Assembling, and Annotation of the Plastid Genomes

The genomic DNA sample of strain TGD was subjected to Genome-seq analysis using the Illumina Next-seq platform. Approximately 127 million of 150 base paired-end reads were generated (38.1 Gb in total). Initial reads were examined by fastQC to filter the reads containing low-quality bases (under 35) more than 20%. We trimmed the adapter sequence and excluded low-quality bases by FASTX toolkit, yielding approximately 82 million reads for the analyses described below.

We reconstructed a circular plastid genome of strain TGD by the two steps described below. As the *de novo* assembling of the 82 million reads was computationally intense, approximately 4 million reads were assembled into 116,551 contigs. TBLASTN search was carried out against the resultant contigs using the amino acid sequences of the plastid-encoded proteins of two green algae, *P. minor* and *Chlorella vulgaris*, as queries (GenBank accession numbers NC_016733 and AB001684). We retrieved 8 contigs, which were approximately 71 Kb in total length, as the tentative plastid genome of strain TGD. 37,646 reads were selected for the second assembling by mapping the 82 million reads on the tentative plastid genome contigs. We connected the resultant contigs by combining the paired-end information and PCR experiments. Finally, a circular DNA molecule of 71,225 bp was reconstructed as the plastid genome of strain TGD. SPAdes ver.3.7.1 (Prjibelski et al., 2020) and Bowtie2 (Langmead and Salzberg, 2012) were used for *de novo* assembling and mapping, respectively.

The genomic DNA sample of strain MGD was shipped to a biotech company (Hokkaido System Science Co., Ltd., Hokkaido, Japan) for Genome-seq analysis using the Illumina Hiseq 2000 platform. Approximately 224 million of 100 bp paired-end reads were generated (33.0 Gb in total). After the quality control (see above), approximately 60 million reads were assembled into 379,981 contigs using Ray (Boisvert et al., 2010). We repeated the TBLASTN search described above and identified a single contig of approximately 100 Kb in length as the plastid genome contig. The PCR experiment using a set of the primers, which were designed based on both edges of the 100-Kb contig (5′-GGGGAGAAATTTCAAGATACGG-3′ and 5′-GGGAGGCAAAGGATAAACTAAACG-3′), amplified a single DNA fragment of approximately 2 Kb in length. We failed to determine the complete nucleotide sequence of the amplicon which is largely composed of the repeats of 84 bp (5′-TTA TTTAATGTCACAAAGCCAAATATATAGGCTTTCTATATGTA GAAAGACCAGTTTATTTAATAAAAAGAATAAATTTTAT GT-3′). We conclude that strain MGD has a circular plastid genome of approximately 102 Kb in length, albeit the exact length of the plastid genome remains uncertain.

We annotated the plastid genomes of strains TGD and MGD as follows. The ORFs encoding polypeptides of equal to or more than 100 amino acid residues were identified by MFannot^1^. Genes encoding transfer RNAs (tRNAs) were surveyed by tRNAscan-SE^2^ (Chan and Lowe, 2019). Ribosomal RNAs, RNase P RNA, and introns were investigated by RNAweasel^3^.

### Detection of Possible RNA Editing

The possibility of RNA editing was explored by comparing RNA-seq reads with the plastid genome sequences. The RNA-seq data of strain TGD (GenBank accession number DRR190720), strain MGD (DRR190721), and *L. chlorophorum* (DRR124369) were downloaded from DDBJ Sequence Read Archive (Kodama et al., 2012). We also downloaded the RNA-seq data of *P. minor* SAG 1965-3 (ERR2041093) generated by the 1000 Plant Transcriptomes Initiative^4^ (Carpenter et al., 2019; One Thousand Plant Transcriptomes Initiative, 2019). After quality control with FASTP v.0.12.4 (Chen et al., 2018), all reads were mapped with HISAT2 v.2.2.1 (Kim et al., 2019) to the corresponding plastid genomes. Besides the two plastid genomes of strains MGD and TGD determined in this study, we used those of *L. chlorophorum* and *P. minor* deposited in the GenBank database under the accession numbers NC_027093.1 and FJ968740.1, respectively. The numbers of RNA-seq short reads subjected to the mapping and those aligned with the corresponding plastid genomes are summarized in Supplementary Table 1.

The incongruities between the RNA-seq reads and plastid genome sequences were detected with mpileup and bcftools commands from bcftools v.1.9 (Li, 2011; Danecek et al., 2021). In this study, we set the two conditions to identify the positions that underwent RNA editing. First, quality-scores of variant calls were greater than 200. Second, the candidate positions are located in ORF/gene coding regions. Only the positions fulfilling both of the two conditions were regarded as post-transcriptional base conversions.

### Phylogenetic Analyses of Plastid-Encoded Proteins

The phylogenetic relationship among green algal plastids and three peDinoflagellate plastids was examined by the maximum-likelihood (ML) phylogenetic analysis of 50 plastid-encoded proteins. The alignment generated by Kamikawa et al. (2015) was modified by adding the plastid-encoded proteins of strains TGD and MGD, as well as three free-living pedinophycean green algae, namely *P. tuberculata*, *Marsupiomonas* sp., and strain YPF701 (Lemieux et al., 2014; Jackson et al., 2018). The amino acid sequences were aligned by MAFFT v.7.490 with L-INS-I option (Katoh, 2002), following manual trimming of ambiguously aligned positions. The final “50-protein” alignment comprised 50 plastid-encoded proteins from 37 taxa (8,736 amino acid positions in total). The 50-protein alignment was subjected to the ML analysis with the LG + Γ + F + C60. Non-parametric bootstrap support values were calculated by 100-replicate ML bootstrap analysis with the LG + Γ + F + C60 + PMSF [posterior mean site frequencies; see Wang et al. (2018)] model. The ML and ML bootstrap analyses described above were repeated after excluding two out of the three peDinoflagellates considered in the original 50-protein alignment. We used IQTREE v.2.1.3 (Nguyen et al., 2015) for both ML and ML bootstrap analyses described above. The alignments generated in this study are available as a part of the Supplementary Data.

We also conducted Bayesian phylogenetic analysis with the CAT + GTR model by using Phylobayes v.1.8a (Lartillot and Philippe, 2004, 2006; Lartillot et al., 2007). Two Markov chain Monte Carlo (MCMC) runs ran for 10,000 cycles and the consensus tree with branch lengths and Bayesian posterior probabilities (BPPs) were calculated after the first one-fourth cycles were discarded as “burn-in.” Note that the maxdiff value stayed large (0.371194) but we found that the trees from the two MCMC chains agreed largely with the ML tree.

### Comparison in Branch Length Between peDinoflagellate and Pedinophycean Green Algae

The 50-protein phylogeny indicated that the branches of the three peDinoflagellates were much longer than those of the four pedinophycean green algae (see section “Results”). We examined the magnitude of “long branch-ness” of the three peDinoflagellates in the individual single-protein alignments. We prepared a 5-taxon tree comprising the four pedinophycean green algae and one of the three peDinoflagellates. The 5-taxon tree was enforced to have the sister relationship between *P. minor* and *P. tuberculata* and that between *Marsupiomonas* sp. and Pedinophyceae sp. YPF-701. The branch lengths of the 5-taxon tree were optimized over each of the 50 single-protein alignments. The branch length optimization was performed by IQTREE v.2.1.3 (Nguyen et al., 2015) with the LG + Γ + F + C60 model.

For each tree, the length of the peDinoflagellate branch was subtracted by the sum of the lengths of the rest of branches to obtain “branch length-ratios.” We split the 50 plastid-encoded proteins into two functional categories, “photosynthetic” and “non-photosynthetic.” The former category contained 30 proteins involved in photosynthesis, while the latter was composed of 17 ribosomal proteins, translation elongation factor Tu, Ycf3, and ClpP. The branch length-ratios of the 30 photosynthetic proteins were compared with those of the 20 non-photosynthetic proteins by Wilcoxon rank-sum test.

## Results

### Overview of the Plastid Genome of the peDinoflagellate Strain TGD

**FIGURE 2:**
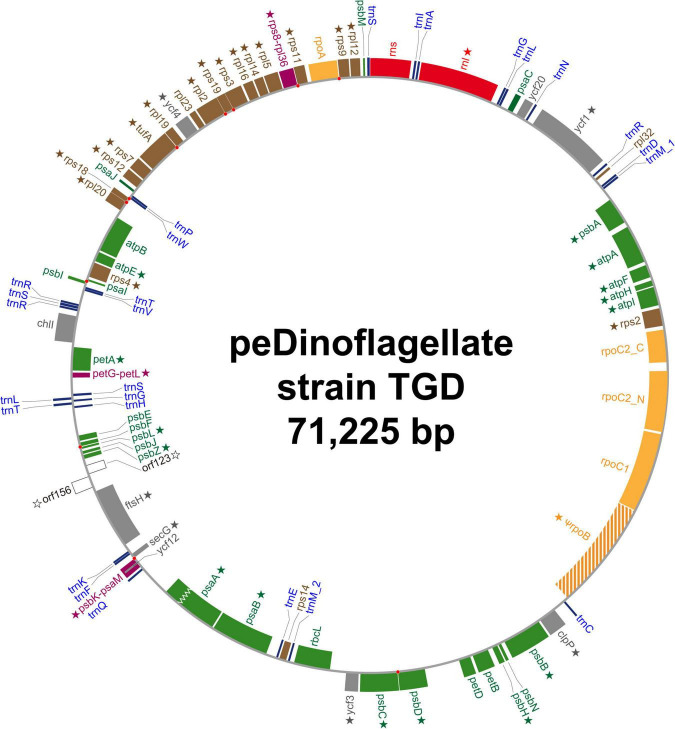
Circular map of the plastid genome of the peDinoflagellate strain TGD. Open reading frames (ORFs) encoding proteins involved in photosynthesis, translation, transcription, and other function are colored in green, brown, orange, and gray, respectively. Functionally unassigned ORFs are shown in white. Fused ORFs are highlighted in purple. *rpoB* in the TGD genome is considered as a pseudogene. The ORF for PsaA in the TGD genome was found to be interrupted by a frameshift (marked by a zigzag line). Ribosomal RNA genes and transfer RNA genes are colored in red and blue, respectively. The ORFs/genes, of which transcripts received RNA editing, are marked by stars. Red dots indicate the overlap of two neighboring ORFs/genes.

We completely sequenced a single, circular DNA molecule of 71,225 bp in length as the plastid genome of the peDinoflagellate strain TGD (TGD plastid genome). The circular genome map is provided as Figure 2 and the general features are summarized in Table 1. The content of guanine plus cytosine (GC content) is 34.8%. 69 ORFs, the genes for small and large subunit rRNA (*rns* and *rnl*), and the genes for 27 tRNAs were identified in the genome. 67 out of the 69 ORFs were functionally assignable. Neither BLAST nor Pfam search provided any clue to the function of *orf123* or *orf156*. By mapping the RNA-seq reads on the nucleotide sequence of the plastid genome, the incongruities of the nucleotide identity were detected between the genome and transcripts at 177 positions (0.0327% of the coding region; Table 2) in 40 ORFs and *rnl* (marked by stars in Figure 2). Thus, we concluded that base conversion editing occurred at the 177 positions in the plastid gene transcripts. The RpoB-coding region was found to be interrupted by a single stop codon in the genome. Likewise, a frameshift hinders the recovery of the continuous PsaA-coding region in the genome (a zigzag line in Figure 2). After referring to the RNA-seq data mapped on the two regions, we regard *rpoB* as a pseudogene while *psaA* is a functional gene. Although *rpoB* seems to be transcribed at a certain level, the stop codon remains in the corresponding transcripts. On the other hand, the single reading frame encoding the entire PsaA was recovered post-transcriptionally by the insertion of two consecutive nucleotides (Supplementary Figure 1). Thus, we conclude that the *psaA* transcript receives base insertion editing and is functional. The RpoC2-coding region was split into two separate ORFs (*rpoC2_N* and *rpoC2_C*; Figure 2) and we detected the distinct transcripts from the two ORFs by analyzing the RNA-seq data. No intron was found in any ORFs identified. Intergenic regions occupy 17.9% of the plastid genome. Three pairs of ORFs, namely (i) *psaM* and *psbK*, (ii) *rps8* and *rpl36*, and (iii) *petL* and *petG* are fused to each other (colored in purple in Figure 2). We had no evidence for any post-transcriptional processing of the transcripts from the three fused ORFs, except for base conversion editing. Noteworthy, the coding region for PsbK-PsaM fused protein was found to encode the entire amino acid sequence of Ycf12 on a different reading frame (Supplementary Figure 2). Ten pairs of neighboring ORFs are on distinct reading frames but partially overlap each other. We found a single case of partial overlapping between a tRNA gene and an ORF. The ORF/gene overlappings described above are highlighted by red dots in Figure 2 (see also Supplementary Table 2 for the details). IRs were not found. One of the three Ile codons in the standard genetic code, AUA, most likely assigns Met (AUA = Met) in the TGD plastid genome, as reported in that of *L. chlorophorum* (Matsumoto et al., 2011).

**TABLE 1:** General features of the plastid genomes in four pedinophycean green algae and three peDinoflagellates.

	*Pedinomonas minor*	*Pedinomonas tuberculata*	*Marsupiomonas* sp. NIES–1824	Pedinophyceae sp. YPF701	*Lepidodinium chlorophorum*	Strain TGD	Strain MGD
GC content (%)	34.8	33.4	40.3	37.7	34.6	34.8	34.6
Genome size (bp)	98,340	126,694	94,262	91,755	66,223	71,225	∼102,000
intergenic regions (%)	25.6	33.8	24.0	27.0	13.3	17.9	37.3
Inverted repeats	+	+	+	+	−	−	−
functionally assignable ORFs	75	74	74	73	61	67	68
rRNA genes	4	6	6	6	2	2	2
tRNA genes	28	28	28	24	27	27	28
Gene containing introns	0	8	0	0	3	0	1
Pseudogenes	0	0	0	0	1	1	1
Gene fusion	0	0	0	0	2	3	4
Gene overlapping	2	1	2	1	11	12	12
Genetic code	Standard	Standard	Standard	Standard	AUA = Met	AUA = Met	AUA = Met
RNA–editing	−	Not examined	Not examined	Not examined	+	+	+
No. of the editing sites					188	177	18
% of the editing sites in the coding sites					0.327%	0.304%	0.0281%

**TABLE 2:** Patterns of base-conversion editing in the plastid gene transcripts of the three peDinoflagellates, *Symbiodinium minutum*, and *Karlodinium veneficum*.

	*Lepidodinium chlorophorum*	Strain TGD	Strain MGD	*Symbiodinium minutum*	*Karlodinium veneficum*
A→U	0	0	1	+	+
A→G	118	88	15	+++	+++
A→C	0	0	0	+	+
U→A	0	1	0	−	+
U→G	0	0	0	+	+
U→C	59	82	0	++	++
G→A	5	0	1	+	+
G→U	0	0	0	−	−
G→C	5	0	0	+	+
C→A	0	0	0	−	+
C→U	1	6	1	++	+
C→G	0	0	0	−	+
Total	188	177	18	389	1,087

*For S. minutum and K. veneficum, the frequency of each base-conversion type is shown by symbols as follows: +++, ≥50%; ++, ≥10%; +, <10%; −, not detected. The corresponding data were coopted from Tables 1, 2 in Klinger et al. (2018). For the three peDinoflagellates, the number of each editing type is shown.*

### Overview of the Plastid Genome of the peDinoflagellate Strain MGD

**FIGURE 3:**
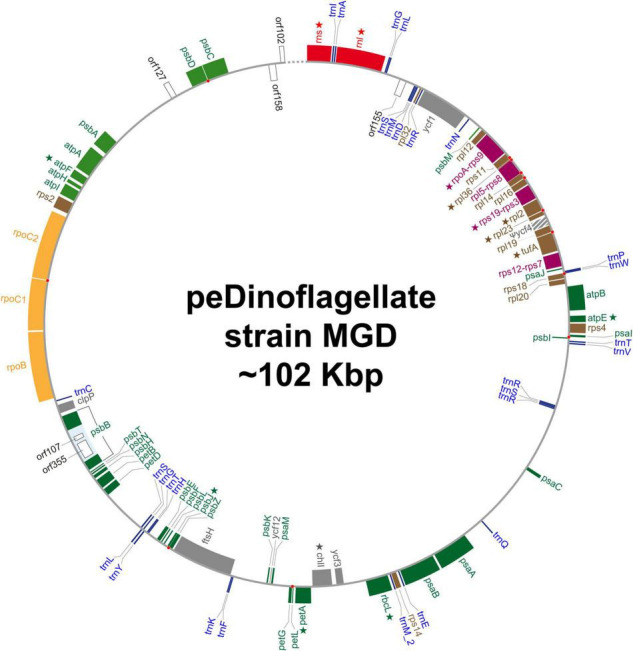
Circular map of the plastid genome of the peDinoflagellate strain MGD. The details of this figure are the same as Figure 1. *ycf4* in the MGD genome is considered as a pseudogene. The precise nucleotide sequence between *orf102* and *rns*, which is indicated by a dotted line in this figure, could not be determined because of repeat sequences. Star indicates the genes of which transcripts receive RNA editing.

We recovered a single, circular DNA molecule of approximately 102 Kb in length as the plastid genome of the peDinoflagellate strain MGD (MGD plastid genome). See Figure 3 and Table 1 for the circular genome map and general features, respectively. The precise length of the plastid genome remains uncertain, as the nucleotide sequence of the region composed of the 84 bp-repeats could not be determined completely. The GC content is 34.6%. Seventy-one ORFs, two intron-encoded ORFs, *rns*, *rnl*, and the genes for 28 tRNAs were annotated in the genome. By mapping RNA-seq reads on the genome, we detected base conversion editing at 18 positions (0.0281% of the coding region; Table 2) in the transcripts of 12 ORFs, *rns*, and *rnl* (marked by stars in Figure 3). No sign of base insertion editing was detected. The functions of three out of the 71 ORFs could not be assigned (i.e., *orf158*, *orf155*, and *orf172*). We regard *ycf4* as a pseudogene (ψ*ycf4*), as the putative N- and C-termini were found to be coded in different reading frames and few transcripts were mapped on this region (Supplementary Figure 3). A single group II intron, which hosts two ORFs (*orf107* and *orf355*), was found in *psbB*. The non-coding region occupies 34.6% of the plastid genome. Four pairs of ORFs, namely *rpoA* and *rps9*, *rpl5* and *rps8*, *rps19* and *rps3*, and *rps12* and *rps7* are fused to each other (colored in purple in Figure 3). No post-transcriptional processing was observed for the transcripts from the fused ORFs, except for base conversion editing that occurred on the *rpoA*-*rps9* and *rpl5*-*rps8* transcripts. Eight pairs of neighboring ORFs are on distinct reading frames but partially overlap each other. We found a single case of partial overlapping between a tRNA gene and an ORF. The ORF/gene overlappings described above are highlighted by red dots in Figure 3 (see also Supplementary Table 2 for the details). IRs were not found. The genetic code used in the MGD plastid genome appeared to be the same as those in the plastid genomes of *L. chlorophorum* and strain TGD (see above).

### Searches for RNA Editing in the Transcripts From the *L. chlorophorum* and *P. minor* Plastid Genomes

Until the RNA-seq data became available (Matsuo and Inagaki, 2018), we could not test whether RNA editing occurs to the plastid gene transcripts in *L. chlorophorum*. In this study, the mapping of the RNA-seq reads on the nucleotide sequence of the *L. chlorophorum* plastid genome revealed base conversion editing at 188 positions (0.327% of the coding region; Table 2) in 28 ORFs, two rRNA genes (*rns* and *rnl*), and isoleucine and asparagine tRNAs (*trnI* and *trnN*). The genes, of which transcripts receive base conversions, are marked by stars in Supplementary Figure 4. *rpoC1* has been regarded as a pseudogene due to multiple in-frame stop codons (Kamikawa et al., 2015). We detected the *rpoC1* transcripts as reported in Kamikawa et al. (2015), albeit the in-frame stop codons remained in the transcript reconstructed from the RNA-seq data.

We applied the above-mentioned analysis to *P. minor* using the plastid genome data and RNA-seq data. Although the amounts of the RNA-seq reads mapped on the plastid genome were compatible among *P. minor* and the three peDinoflagellates (Supplementary Table 1), no case of RNA editing was detected in the transcripts from the *P. minor* plastid genome.

### Origin(s) of the TGD and MGD Plastids Inferred From a 50-Protein Phylogeny

**FIGURE 4:**
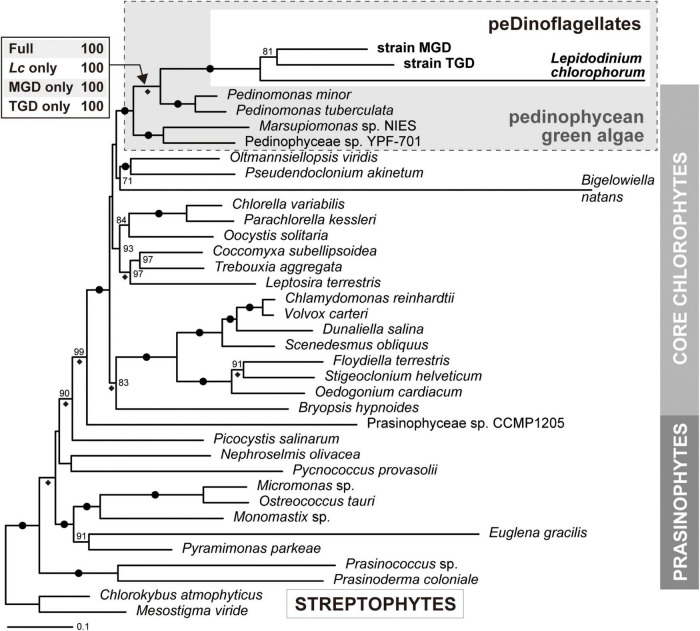
Phylogeny of green algal plastids and green alga-derived plastids inferred from the alignment comprising 50 plastid-encoded proteins. The maximum-likelihood (ML) tree and non-parametric ML bootstrap support values (MLBP) are shown here. The overall tree topology inferred from Bayesian method agreed with the ML tree. Dots on branches indicate the bipartitions received MLBPs of 100% and Bayesian posterior probabilities (BPPs) of 1.0. Only MLBPs greater than 70% are shown. The bipartitions supported by BPPs of 1.0 are marked with diamonds. For the node uniting the three peDinoflagellates and pedinophycean green algae, the MLBPs calculated from the four ML bootstrap analyses are presented. “Full,” the analysis considering the three peDinoflagellates; “*Lc* only,” “MGD only,” and “TGD only,” those considering *Lepidodinium chlorophorum*, strain MGD, and strain TGD considering the sole representative of peDinoflagellates, respectively.

The origin(s) of the plastids of peDinoflagellate strains TGD and MGD were examined by both ML and Bayesian phylogenetic analyses of an amino acid alignment comprising 50 plastid-encoded proteins. Regardless of the method for tree reconstruction, the monophyly of TGD, MGD, and *L. chlorophorum* was reconstructed (an MLBP of 100% and a BPP of 1.0; Figure 4). The clade of the peDinoflagellates was then nested within pedinophycean green algae with a specific affinity to *Pedinomonas* spp. The above-mentioned relationships received non-parametric ML bootstrap support values (MLBPs) of 100% and BPPs of 1.0 (Figure 4). Furthermore, the sampling of the peDinoflagellates in the ML analyses gave no impact on the affinity between the peDinoflagellates and *Pedinomonas* spp. (Figure 4).

### Difference in Evolutionary Tempo Between Photosynthetic and Non-photosynthetic Plastid-Encoded Proteins in the Three peDinoflagellates

**FIGURE 5:**
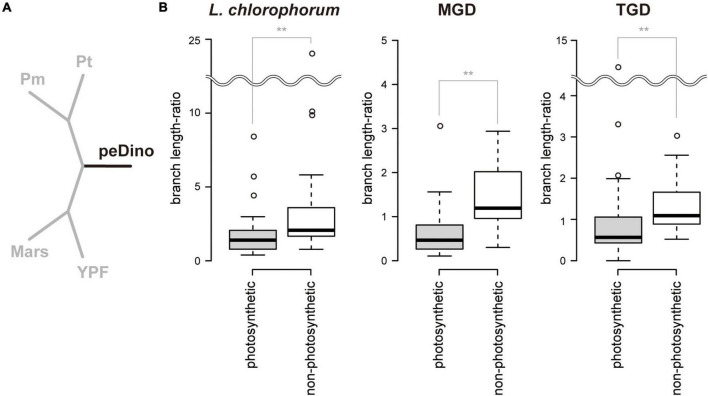
Comparison of branch length between peDinoflagellates and pedinophycean green algae. **(A)** The 5-taxon tree. The branch lengths were optimized based on each of the 50 single-protein alignments. We fixed the relationship among *Pedinomonas minor* (Pm), *Pedinomonas tuberculata* (Pt), *Marsupiomonas* sp. (Mars), Pedinophyceae sp. YPF-701 (YPF), and one of the three peDinoflagellates (peDino). For each tree with the optimum branch lengths, the length of the black branch was subtracted by the sum of the lengths of the grey branches (“branch length-ratio”). **(B)** Box plots of branch length-ratios between 30 “photosynthetic” proteins and 20 “non-photosynthetic” proteins. The ratios calculated based on the 5-taxon trees considering *Lepidodinium chlorophorum*, strain MGD, and strain TGD are shown left, center, and right, respectively. Wilcoxon rank-sum test rejected the null hypothesis of no difference in the median values of branch length-ratios calculated from the two categories was rejected with *p* < 0.01 (highlighted by double asterisks). The 5-taxon trees with the optimum branch lengths and branch length-ratios are provided as Supplementary Material.

The branches of the three peDinoflagellates in the 50-protein phylogeny were found to be longer than those of the pedinophycean green algae (Figure 4). The ratios of the branch length for a peDinoflagellate to those of the four pedinophycean green algae (branch length-ratios) were calculated for each protein considered in the alignment, aiming to identify the plastid-encoded proteins that contributed to the long branch-ness observed in the 50-protein phylogeny (Figure 5A). The peDinoflagellate branches were generally long in the vast majority of the single-protein trees, regardless of the peDinoflagellate included (see Supplementary Material). Nevertheless, the branch length-ratios calculated from the non-photosynthetic proteins tend to be larger than those from the photosynthetic proteins (Figure 5B). The Wilcoxon rank-sum test rejected the null hypothesis of no difference between the median values of the branch length-ratios calculated from the two categories at the 1% level (*p* = 7.50 × 10^–3^, 3.49 × 10^–5^, and 2.14 × 10^–3^ in the comparisons considering *L. chlorophorum*, strain MGD, and strain TGD, respectively). These results suggest that the overall substitution rates in non-photosynthetic plastid-encoded proteins are higher than those in photosynthetic plastid-encoded proteins in the three peDinoflagellates. The significant difference in substitution rate between photosynthetic and non-photosynthetic plastid-encoded proteins may have been achieved separately in *L. chlorophorum*, strain MGD, and strain TGD.

### Features Shared Among the peDinoflagellate Plastid Genomes but Not Found in the Pedinophycean Plastid Genomes

Prior to the comparison between the plastid genomes of the three peDinoflagellates and those of pedinophycean green algae, we briefly review the four pedinophycean plastid genomes sequenced completely to date (Table 1). As do phylogenetically diverse algae, the four pedinophycean green algae have the plastid genomes with low GC content (33.4–40.3%) and IRs (Table 1). Although the sizes of the four plastid genomes vary from 91.8 to 126.7 Kb, they appeared to carry similar numbers of functionally assignable ORFs (73–75; henceforth here designated simply as “ORFs”). The plastid genome of *Pedinomonas tuberculata* (126.7 Kb) appeared to be larger than the rest of the three plastid genomes (91.8–98.3 Kb) compared here, partially due to 7 introns in the *P. tuberculata* plastid genome but none in others (Table 1).

We revealed 7 of the plastid genome features that were shared among the three peDinoflagellates but were not found or much less obvious in the pedinophycean green algae. (i) The peDinoflagellate plastid genomes commonly lack IRs (Figures 2, 3, and Supplementary Figure 3), although the four pedinophycean plastid genomes bear IRs (Table 1). (ii) We identified more than 10 cases of ORF/gene overlapping/fusion in the peDinoflagellate plastid genomes (Table 1). In contrast, only a few cases of ORF overlapping and no ORF fusion were detected in the four pedinophycean plastid genomes. (iii) The peDinoflagellate plastid genomes appeared to carry fewer numbers of ORFs (i.e., 61–68) than 73–75 ORFs found in those of the four pedinophycean green algae (Table 1). The ORF repertories of the three peDinoflagellate plastid genomes appeared to be similar to each other but the TGD and MGD plastid genomes retain 6–8 ORFs that are absent from the *L. chlorophorum* plastid genome (Figure 5). (iv) The peDinoflagellate plastid genomes commonly use a deviant genetic code, in which AUA is assigned as methionine, not as isoleucine as the standard genetic code (Table 1). (v) Certain levels of pseudogenization seemingly operated on the peDinoflagellate plastid genomes. A single non-functional ORF was found in the individual peDinoflagellate plastid genomes, albeit none has been reported in the pedinophycean plastid genomes. (vi) Base conversion type RNA editing appeared to occur to the plastid gene transcripts in the three peDinoflagellates (see below). Importantly, we found no evidence for RNA editing on the transcripts from the *P. minor* plastid genome. Finally, (vii) the significant difference in substitution rate between the plastid-encoded proteins in “photosynthetic” category and those in “non-photosynthetic” category appeared to be shared among the three peDinoflagellates (Figure 5B).

### Patterns and Frequencies in Base Conversion RNA Editing on the Transcripts From the Three peDinoflagellate Plastid Genomes

Base conversion editing on plastid gene transcripts was observed in the three peDinoflagellates (the details of the sites received base conversion editing are summarized in a spreadsheet in Supplementary Data). However, the pattern and frequency of RNA editing appeared to vary among the three peDinoflagellate plastids. We observed similar numbers of editing positions in the *L. chlorophorum* and TGD plastids (188 and 177). In the two peDinoflagellate plastids, more than 90% of the identified editings converted A to G or U to C (Table 2). In the *L. chlorophorum* plastid, three minor types of base conversion, namely conversion from G to A, that from G to C, and that from C to U, were observed. We observed a single case of the conversion from U to A and 6 cases of the conversion of C to U in the TGD plastid.

The trend of the editing in the MGD plastid appeared to be distinct from those in the *L. chlorophorum* and TGD plastids described above in two aspects. First, only 18 editing positions were detected in the MGD plastid (Table 2). Second, the conversion from A to G occupied 15 out of the 18 observed editing positions but no case of the conversion from U to C was found (Table 2). The rest of the editings converted A to U, G to A, and C to U.

The majority of the base conversion editing in the transcripts from protein-coding regions in the three peDinoflagellate plastids were found at codon first and second positions. In *L. chlorophorum*, 86, 60, and five cases of the editing were found at codon first, second, and third positions, respectively, after exclusion of those occurred in tRNAs, rRNAs, and the regions overlapped by two ORFs (Table 3). A similar strong bias of the editing toward codon first/second positions over third positions was observed in the TGD and MGD plastids (Table 3).

**TABLE 3:** Frequencies of base conversion-type RNA editing at the codon positions in the three peDinoflagellates.

	*Lepidodinium chlorophorum*	Strain TGD	Strain MGD
Codon first position	86	81	10
Codon second position	60	80	2
Codon third position	5	12	0
Regions overlapped by two ORFs	3	2	1
Transfer and ribosomal RNAs	34	3	5

A single editing position in the *petA* transcript appeared to be shared between the *L. chlorophorum* and TGD plastids. A-to-G conversions occurred at the homologous positions in the *L. chlorophorum* and TGD *petA* transcripts (Supplementary Figure 5). These editings introduced the amino acid change from lysine to arginine and that from aspartic acid to glycine in the *L. chlorophorum* and TGD proteins, respectively. All editings except the case described above were found to occur at unique positions across the three peDinoflagellate plastids.

## Discussion

### Reconfirmation of the Pedinophycean Origin of the *L. chlorophorum*, MGD, and TGD Plastids

The plastid phylogeny inferred from plastid small subunit rRNA genes demonstrated the pedinophycean green algal origin of the *L. chlorophorum*, TGD, and MGD plastids (Sarai et al., 2020). In the current study, we strengthened the pedinophycean green algal origin of the three peDinoflagellate plastids by analyzing the 50-protein alignment (Figure 4). The *L. chlorophorum*, TGD, and MGD plastids were grouped together and connected to the *P. minor* and *P. tuberculata* plastids with full statistical support. At the face value, the 50-protein phylogeny indicates that the three dinoflagellates took up the same pedinophycean green alga as the endosymbionts. However, we had a suspicion of the three peDinoflagellate plastids being grouped together artifactually and misplaced in the tree of green algal plastids, as the proteins encoded in the three peDinoflagellate plastids appeared to evolve much more rapidly than the orthologs in the green algal plastids.

To examine the above-mentioned possibility, we reanalyzed the 50-protein alignment after excluding two out of the three peDinoflagellate plastids alternatively. Significantly, the 50-protein phylogeny constantly grouped the *Pedinomonas* plastids and one of the three peDinoflagellate plastids together, arguing against their intimate phylogenetic affinity being an artifact in the tree reconstruction. The conclusion deduced from the 50-protein analyses agrees well with the discussion in Sarai et al. (2020)—*L. chlorophorum*, strains MGD, and strain TGD separately transformed green algal endosymbionts belonging to the genus *Pedinomonas* or those closely related to *Pedinomonas*. Nevertheless, there is a large possibility for the endosymbionts in the three peDinoflagellates being closely related but different species belonging to the genus *Pedinomonas*, as the genuine diversity of *Pedinomonas* (and their close relatives) is underrepresented by *P. minor* and *P. tuberculata*, for which plastid genome data are currently available. The phylogenetic relationship among the three peDinoflagellate plastids should be reexamined after we obtain the plastid genome data from the species that sufficiently represent the diversity of pedinophycean green algae in the natural environment.

In order to address why the pedinophycean endosymbiosis was repeated in distantly related branches in the tree of dinoflagellates, we need to accumulate both environmental and physiological data of pedinophycean green algae and peDinoflagellates. First, the three peDinoflagellates are of marine and most likely took up marine pedinophycean algae as the endosymbionts. Nevertheless, *P. minor* and *P. tuberculata* were isolated originally from fresh water and soil environments, respectively. Thus, future studies may explore the diversity and distribution of *Pedinomonas* and their close relatives in the marine environment. Secondly, the repeated pedinophycean endosymbiosis imply the potential merits for the host cells to bear the pedinophycean-derived plastids in their natural habitats. Thus, it is necessary to examine the key factors for the emergence of peDinoflagellates in the marine environments and the physiological characteristics that differentiate peDinoflagellates from other dinoflagellate species.

### Unique Features Shared Among the Three peDinoflagellate Plastid Genomes: Parallel Gain or Vertical Inheritance?

The comparative plastid genomics identified the features that are common in the three peDinoflagellate plastids but absent in the pedinophycean plastids (see section “Results”). We here propose that some of the above-mentioned features were achieved in the three peDinoflagellate plastids in parallel.

#### Secondary Loss of Inverted Repeats

The plastid genomes lacking IRs have been documented in diverse land plants (Palmer and Thompson, 1981, 1982; Guisinger et al., 2011; Wu et al., 2011; Li et al., 2016; Ruhlman et al., 2017; Choi et al., 2019; Cauz-Santos et al., 2020; Jin et al., 2020), green algae (Turmel et al., 2009a; Cai et al., 2017), haptophytes (Baurain et al., 2010; Gabrielsen et al., 2011), euglenids (Hallick et al., 1993; Gockel and Hachtel, 2000; Karnkowska et al., 2018), and cryptophytes (Donaher et al., 2009; Tanifuji et al., 2020). As the four pedinophycean plastid genomes determined to date bear IRs, we proposed that the plastid genomes in the pedinophycean endosymbionts taken up by *L. chlorophorum*, strain MGD, and strain TGD used to have IRs but the parallel losses of one of the two copies occurred during serial secondary endosymbioses.

#### Reduced Open Reading Frame Repertory, Pseudogenization, and Difference in Evolutionary Tempo Between Photosynthetic and Non-photosynthetic Proteins

**FIGURE 6:**
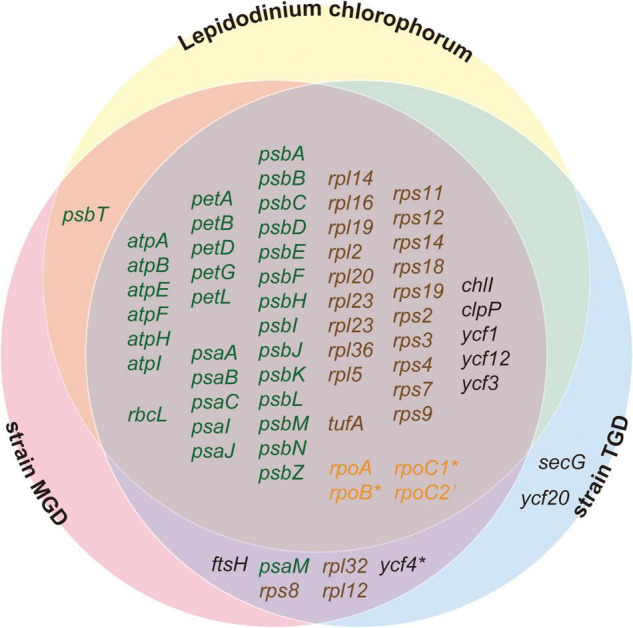
Venn diagram for the functionally assignable ORFs in the three peDinoflagellate plastid genomes. Open reading frames (ORFs) encoding proteins involved in photosynthesis, translation, transcription, and other function are colored in green, brown, orange, and black, respectively. Notes, *rpoC1* in *Lepidodinium chlorophorum*, *rpoB* in strain TGD, and *ycf4* in strain MGD are pseudogenes and marked by asterisks. *rpoC2* is highlighted by an exclamation mark as the N- and C-termini of RpoC2 are encoded in two distinct ORFs in strain TGD (see Figure 1).

Uthanumallian et al. (2022) recently demonstrated the parallel reduction of ORF repertory in the plastid genomes in chlorarachniophytes, euglenids, and *L. chlorophorum*, all of which established their current plastids throughout the reductions of green algal endosymbionts. By expanding the discussion in the pioneering work, we propose the parallel reduction of ORF repertoires in the *L. chlorophorum*, TGD, and MGD plastid genomes. The convergence of ORF repertoires among the three peDinoflagellate plastid genomes (Figure 6) can be reconciled if the pressure for discarding the genes encoding the proteins not involved in the core plastid functions, such as photosynthesis, translation, and transcription (Uthanumallian et al., 2022), was common across serial secondary endosymbioses. Pseudogenization can be regarded as a part of the putative parallel reductions of ORF repertory during serial secondary endosymbioses.

Our inspection revealed that non-photosynthetic proteins evolved more rapidly than photosynthetic proteins in the three peDinoflagellate plastids (Figure 5B). This feature likely shaped the difference in ORF repertory among the three peDinoflagellate plastids. *L. chlorophorum* has the most compact plastid genome among the three peDinoflagellates and may have lost at least extra 8 genes comparing to strains TGD and MGD (Figure 6). Interestingly, the genes lost from the *L. chlorophorum* plastid genome contain 7 “non-photosynthetic” genes (including *rpl12*, *rpl32*, and *rps8* encoding ribosomal proteins) and only a single “photosynthetic” gene encoding a component of photosystem I (*psaM*). The non-photosynthetic-versus-photosynthetic ratio in the genes lost from the *L. chlorophorum* plastid genome may reflect the difference in evolutionary tempo between the two functional categories of plastid-encoded proteins.

#### Open Reading Frame/Gene Overlapping/Fusion

All the cases of ORF/gene overlapping/fusion found in the three peDinoflagellate plastid genomes unlikely occurred independently. At least, the overlapping of *psbD* and *psbC* is shared among the four pedinophycean and three peDinoflagellate plastid genomes (Supplementary Table 2), suggesting that the two genes have already been overlapped in the plastid genomes of the pedinophycean endosymbionts taken up by the dinoflagellate hosts. *petL* and *petG* were found to be fused or overlapped with each other in the three peDinoflagellate plastid genomes (Supplementary Table 2), albeit the homologous genes in all of the four pedinophycean plastid genomes are next to but separated from each other (Turmel et al., 2009b; Jackson et al., 2018). Thus, it is difficult to favor absolutely one of the following two possibilities over the other, one assuming that the overlapping/fusion of *petL* and *petG* has been descended from the plastid genomes of the endosymbionts and the other assuming that the two neighboring ORFs were overlapped/fused to each other separately during serial secondary endosymbioses.

The MGD and TGD plastid genomes appeared to share six cases of ORFs overlapping/fusion (Supplementary Table 2). This observation prompts us to propose that strains MGD and TGD established their current plastids from two closely related pedinophycean endosymbionts, both of which plastid genomes had the six cases of ORF overlapping/fusion of matter. Alternatively, it is also possible that the ORF overlapping/fusion occurred convergently between the MGD and TGD plastid genomes by combining the two following insights from the comparative analyses of the three peDinoflagellate plastid genomes. First, the MGD and TGD plastid genomes share large syntenic regions (Supplementary Figure 6). Second, the three peDinoflagellate plastid genomes may have been under certain levels of pressure to make neighboring ORFs/genes overlap/fuse to one another, as 6–9 unique cases of ORF/gene overlapping/fusion in each plastid genome (Supplementary Table 2). Thus, the chances of the same pairs of ORFs being overlapped/fused together separately in both MGD and TGD plastid genomes may be considerably high. In sum, it is hard to make any concrete inference on the evolutionary processes worked behind ORF/gene overlapping/fusion found in the three peDinoflagellate plastid genomes, except *psbD-psbC* overlapping shared strictly among the pedinophycean and peDinoflagellate plastids (see above).

#### AUA Codon Reassignment

According to the codon capture hypothesis (Osawa and Jukes, 1989; Matsumoto et al., 2011), we can predict the process, which reassigned AUA codon from Ile to Met in a plastid genome, as follows. First, AUA codons disappeared from the genome by synonymous mutations to either of the standard Ile codons AUU or AUC. Second, the particular Ile tRNA (tRNA*^Ile^*), which was cognate to AUA codon, was discarded from the translation system. Third, a new Met tRNA (tRNA*^Met^*), which can read AUA codon, emerged. Importantly, the disappearance and emergence of the two tRNA species described above were neutral because of the absence of AUA codon in the genome assumed in this hypothesis. After the above-mentioned steps, AUA can have reemerged as a Met codon in the genome. There are alternative scenarios that demand no strict neutrality for each step of codon reassignment (Schultz and Yarus, 1994; Santos et al., 1999; Sengupta and Higgs, 2005; Mühlhausen et al., 2016). Regardless of the level of neutrality during the reassignment of AUA codon, the complex interplay of the change in codon usage and the evolution of tRNAs is required and only a few cases of deviant genetic codes in plastid genomes have been reported prior to this study (Matsumoto et al., 2011; Su et al., 2019; Ceriotti et al., 2021). Considering the number of the plastid genomes completely sequenced to date (6,661 genomes labeled as “apicoplast,” “chloroplast,” or “cyanelle” in the GenBank Genome database as of December 2021), we can regard the reassignment of a codon (or codons) as rare events in the evolution of plastid genomes. We here propose that as-yet-unstudied pedinophycean species possess the plastid genomes, in which AUA codon is assigned as Met, and were the origins of the current peDinoflagellate plastids. In other words, we anticipate the AUA assignment in the plastid genome as the probe to pinpoint the origins of the pedinophycean green algae that gave rise to the three peDinoflagellate plastids.

#### RNA Editing

The original works reported the four pedinophycean plastid genomes did not examine the presence/absence of RNA editing experimentally (Turmel et al., 2009b; Jackson et al., 2018), most likely because the plastid gene sequences and corresponding amino acid sequences lacked any sign of RNA editing. Indeed, the mapping of RNA-seq reads on the plastid genome found no incongruence between the genome and transcript sequences in the *P. minor* plastid by our criterion (see above). Thus, until a future study finds a clear case of RNA editing in an as-yet-unstudied pedinophycean plastid, we here propose that (i) the pedinophycean plastids are primarily free from RNA editing and (ii) the three peDinoflagellates configured and started executing the RNA editing on the plastid gene transcripts in parallel.

One may argue that the machinery required for RNA editing was too complex to evolve *de novo* in the three separate occasions in the evolution of dinoflagellates. It is worthy to note that RNA editing has been documented in peridinin plastids of diverse dinoflagellates, suggesting that this molecular trait can be traced back to an early dinoflagellate species bearing peridinin plastid (Zauner et al., 2004; Dang and Green, 2009; Dorrell and Howe, 2012; Mungpakdee et al., 2014; Klinger et al., 2018). Thus, the ancestral species, which gave rise to *L. chlorophorum*, strain MGD, and strain TGD, used to bear peridinin plastids and most likely operated the RNA editing on plastid gene transcripts. We propose that *L. chlorophorum* and strain TGD transplanted the RNA editing machinery, which originally worked in peridinin plastids, to the plastids derived from the pedinophycean endosymbionts. The principal reason for the above proposal is the similarity between the pattern of base conversion editing between *L. chlorophorum*/TGD and peridinin dinoflagellates. In both peridinin and two peDinoflagellate plastids, base conversion occurred in diverse directions, but the cases of A-to-G and U-to-C conversions appeared to predominate over other types of base conversion (Table 2). The above proposal is not totally unexpected, as the putative co-option of the RNA editing machinery in peridinin plastid was proposed for the non-canonical plastids derived from the haptophyte endosymbiont in the ancestral kareniacean dinoflagellate for the same reasoning (Dorrell and Howe, 2012; Jackson et al., 2013; Klinger et al., 2018).

The pattern of the base conversion editing distinguishes the MGD plastid from other dinoflagellate plastids including those of *L. chlorophorum* and strain TGD. Due to the absence of U-to-C conversion in the 18 editing positions identified in the MGD plastid (Table 2), we have no solid ground to assume that base conversion editing in the MGD plastid is performed by the machinery that existed prior to serial secondary endosymbiosis. One possibility is that strain MGD has retained the RNA editing machinery beyond serial secondary endosymbiosis but discarded the molecular components that were required for U-to-C conversion.

We here explore why the pattern and frequency of base conversion editing are different between strain MGD and *L. chlorophorum*/strain TGD, besides the potential difference in the RNA editing machinery (see above). Intriguingly, the plastid-encoded RNA polymerases in both *L. chlorophorum* and strain TGD, of which plastid gene transcripts receive base conversion editing at higher frequencies than those of strain MGD, are potentially deficient. Both *rpoC1* in *L. chlorophorum* and *rpoB* in strain TGD can be regarded as non-functional (Supplementary Figure 4 and Figure 2). In addition, RpoC2 in strain TGD are encoded in two separate ORFs (Figure 2) and it is difficult to exclude absolutely the possibility of this unusual RpoC2 being dysfunctional/non-functional. In contrast, the genes encoding RNA polymerase subunits in the MGD plastid genome seem to be intact (Figure 3). Thus, the potential deficiency in the plastid-encoded RNA polymerase might connect with the frequency of base conversion editing on the corresponding plastid gene transcripts, albeit we can provide no molecular background for the above hypothesis. Curiously, *Karlodinium veneficum*, of which *rpoB* and *rpoC2* were found to be disrupted by frameshifts, operates base conversion editing on plastid gene transcripts at a much higher frequency than *L. chlorophorum* or strain TGD (Gabrielsen et al., 2011; Klinger et al., 2018). There is a large room for the “connection” between the deficiency in plastid-encoded RNA polymerase and frequency of base conversion editing being coincident but worthy to being revisited when the data of genomes and RNA editing are accumulated from additional non-canonical-types of dinoflagellate plastids.

Finally, we found a single case of base insertion editing in the *psaA* transcript in strain TGD (Supplementary Figure 1). This type of RNA editing has not been documented in any dinoflagellate plastids, suggesting that strain TGD has developed this type of RNA editing after serial secondary endosymbiosis. There are three possibilities for the origin of the putative molecular machinery for base insertion editing found in the TGD plastid. First, strain TGD invented the machinery *de novo*. Second, strain TGD laterally acquired the machinery from a distantly related organism. Third, strain TGD modified the machinery for base conversion editing to operating base insertion editing. To evaluate the three scenarios described above, we first need to identify the enzymes that operate the base insertion in the *psaA* transcript in the TGD plastid.

## Conclusion

In the current study, we reported the plastid genomes of peDinoflagellate strains MGD and TGD and unveiled the plastid genome features, many of which were predicted to have emerged separately after serial secondary endosymbioses involved in pedinophycean green algae. Among the features found in the peDinoflagellate plastid genomes, RNA editing is intriguing. The plastid gene transcripts of *L. chlorophorum* and strain TGD appeared to share the pattern of base conversion editing with those of peridinin dinoflagellates, suggesting that the RNA editing machinery was inherited in the two peDinoflagellates beyond serial secondary endosymbioses. On the other hand, we could provide no plausible idea of how strain MGD established the RNA editing in the plastid, as the pattern of base conversion editing on the plastid gene transcripts was distinct between strain MGD and other dinoflagellates including *L. chlorophorum* and strain TGD.

## Data Availability Statement

The nucleotide sequences of the MGD and TGD plastid genomes are available under the GenBank/DDBJ/EMBL accession numbers LC716140 and LC716139, respectively.

## Author Contributions

CS, KT, and MI conducted the experiments related to the algal cultures. EM and KM conducted the molecular biological experiments required for sequencing the plastid genomes. EM, KM, and TN reconstructed the plastid genome sequences and identified RNA editing by mapping RNA-seq reads. EM, EY, and YI prepared alignments and carried out phylogenetic analyses. EM, KM, TN, MI, and YI wrote the manuscript. All authors contributed to the article and approved the submitted version.

## Conflict of Interest

The authors declare that the research was conducted in the absence of any commercial or financial relationships that could be construed as a potential conflict of interest.

## Publisher’s Note

All claims expressed in this article are solely those of the authors and do not necessarily represent those of their affiliated organizations, or those of the publisher, the editors and the reviewers. Any product that may be evaluated in this article, or claim that may be made by its manufacturer, is not guaranteed or endorsed by the publisher.

## Footnotes

1
http://megasun.bch.umontreal.ca/cgi-bin/mfannot/mfannotInterface.pl

2
http://lowelab.ucsc.edu/tRNAscan-SE/

3
https://megasun.bch.umontreal.ca/cgi-bin/RNAweasel/RNAweaselInterface.pl

4
http://www.onekp.com/samples/list.php
