# Supplementary material for: Comparative Plastid Genomics of Green-Colored Dinoflagellates Unveils Parallel Genome Compaction and RNA Editing
Source: Front Plant Sci. 2022 Jul 11;13:918543. doi: 10.3389/fpls.2022.918543 (PMC9309888; doi:10.3389/fpls.2022.918543)
Supplement: Supplementary file 3 [file Data_Sheet_3.PDF]

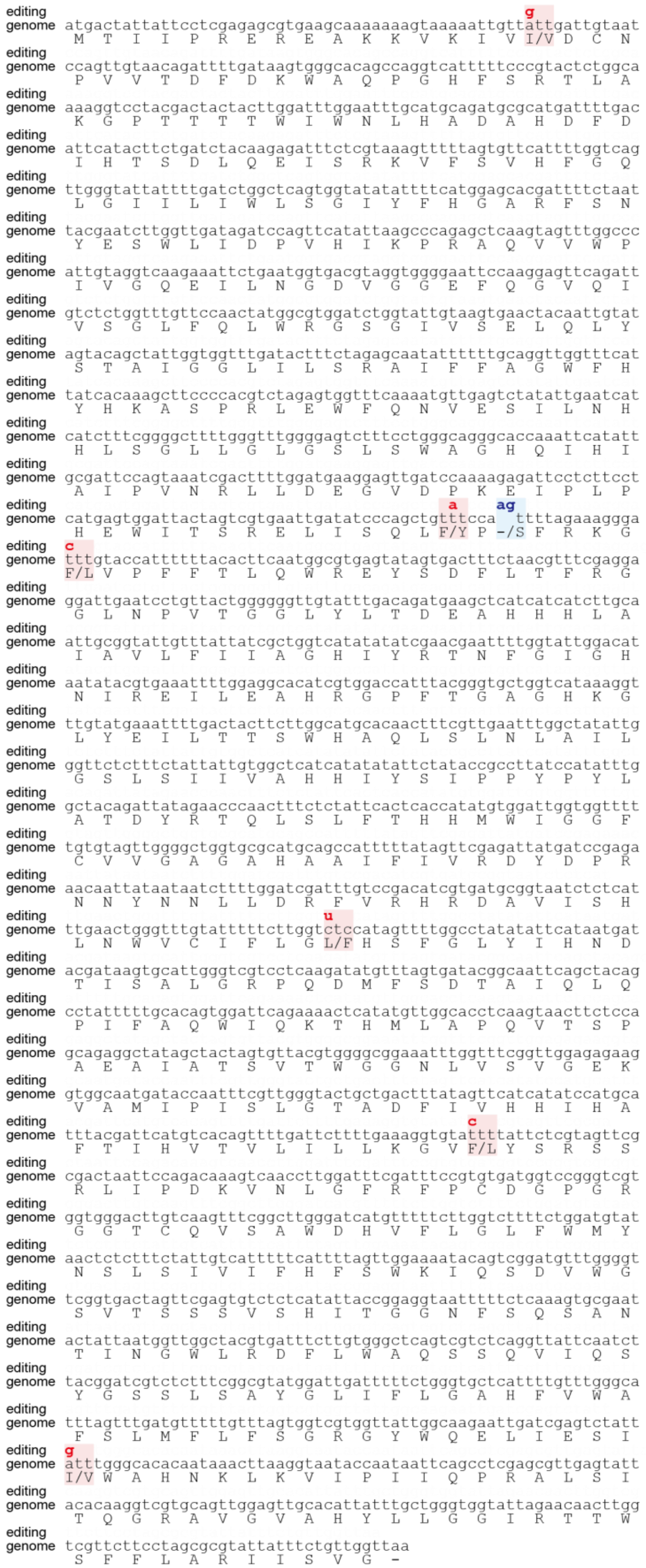

Supplementary Figure 1. RNA editing found in the *psaA* transcript in strain TGD. The nucleotides after RNA editing are indicated above the genome sequence. The letters in red and blue are the results of base-conversion and base-insertion editing, respectively. The amino acid changes led by RNA editing are shaded in red or blue.

```
    atgatctctatcctagtaaaacttcctgaagcatatagaccatttaacccaattgtagat
+1   M  I  S  I  L  V  K  L  P  E  A  Y  R  P  F  N  P  I  V  D

    gtaatacctgttattcccgtatttttctactactagcattcgtatggcaagcgtcggta
+1   V  I  P  V  I  P  V  F  F  L  L  L  A  F  V  W  Q  A  S  V

    agtttccgattagaaaaagttaaaataacgctaagtaaatttttttctttatattattat
+1   S  F  R  L  E  K  V  K  I  T  L  S  K  F  F  S  L  Y  Y  Y

    tattatggaggttattttccaacttgcagctattttcgctgttgtaactgtaggaccagc
+1   Y  Y  G  G  Y  F  P  T  C  S  Y  F  R  C  C  N  C  R  T  S
+2       M  E  V  I  F  Q  L  A  A  I  F  A  V  V  T  V  G  P  A

    agtaatcattcttttggcagcacaaaaagggaatctttatttttttcttatgaatatt
+1   S  N  H  S  F  G  S  T  K  R  E  S  F  I  F  F  L  M  N  I
+2  V  I  I  L  L  A  A  Q  K  G  N  L  L  F  F  F  L  *

    tcggataaccaagtctttatagctctatacgttgcactccttacaggtataatagctctc
+1   S  D  N  Q  V  F  I  A  L  Y  V  A  L  L  T  G  I  I  A  L

    cgtctaggcattaatttgtatgaaggtttaacattaatttttacatgctctta
+1   R  L  G  I  N  L  Y  E  G  L  T  L  I  F  T  C  S  *
```

Supplementary Figure 2. Organization of *psbK*, *psaM* and *ycf12* in the TGD plastid genome. PsbK-PsaM fusion protein is encoded in +1 reading frame. The amino acid residues in red and purple correspond to the conserved domain for PsbK superfamily (PRK02553) and that for PsaM superfamily (CHL00190), respectively. The putative amino acid sequence of Ycf12 is most likely encoded in the same genome region but on a different frame (+2). The amino acid residues in blue correspond to the conserved domain for PSII_Ycf12 superfamily (CHL00184).

A

```
atgattcgtcgagacatcatacttgggccaagacaaactagtaactatttttttttcaga
 M  I  R  R  D  I  I  L  G  P  R  Q  T  S  N  Y  F  F  F  R
aatctatttttggaggaacaatcttttcctcattgggtttccaaattttaggaaaaat
 N  L  F  F  G  G  T  I  F  F  L  I  G  F  P  N  F  R  K  N
ctattgcctaaagctatagaaattaaatttctaccccaaggtatcgttatatgcttttat
 L  L  P  K  A  M  E  I  K  F  L  P  Q  G  I  V  I  C  F  Y
ggtaggttggcaataagattcagtttgtatggggttattagaagatttttgttgattggg
 G  R  L  A  M  R  F  S  L  Y  G  V  I  R  R  F  L  L  I  G
agaggagttaatgagtataataaaaaaaaagcaaaattcatattcttcgttggggatttc
 R  G  V  N  E  Y  N  K  K  K  A
                        K  K  S  K  I  H  I  L  R  W  G  F
ctaacaaaattcgacgaatagaatttcttatttgctttctgaacttgaatcgatcgggt
P  N  K  I  R  R  M  E  F  S  Y  L  L  S  E  L  E  S  I  G
tggtaaatcaaaaacaattgcttcagcctattgaattgaaaatatatttctttgaaag
L  V  N  Q  K  Q  L  L  Q  P  I  E  L  K  M  Y  F  L  L  K
atcgacgaaaaattatttttttgaaacctaatatagaaaatattagctcgctagaagaaa
D  R  R  K  I  I  F  L  K  P  N  M  E  N  I  S  S  L  E  E
tagaacaattttcttctaacttggcgaagtttctacaaattcctttgaaagaagaattac
M  E  Q  F  S  S  N  L  A  K  F  L  Q  I  P  L  K  E  E  L
ccttcgatttttttctttattttcattaagtga
P  F  D  F  F  F  I  F  I  K  -
```

B

```
MGD ψYcf4       M---------------IRRDIILGPRQTSNYFFFRNLFFGGTIFFLIGFP-NFRKNLLP-
P. minor Ycf4   MMSKENFQTNNVTIDNIRRDLVIGSRRFSNYWWACVLSFGGIGFLLTGISSKVQTNLLPF

MGD ψYcf4       -KAMEIKFLPQGIVMCFYGRLAMRFSLYGVIRRFLLIGRGVNEYNKKKSKIHILRWGFPN
P. minor Ycf4   INYQDIQFFPQGLVMSFYGIIALVLSLYLWACIAWSVGGGFNEFNKKDGIVRIFRWGFPG

MGD ψYcf4       KIRRIEFSYLLSELESIGLVNQKQLLQPIELKMYFLLKDRRKIIFLKPNMENISSLEEME
P. minor Ycf4   KNRRIELVYSLNEIDCIK-VDLQEGLNP-RRSIYLRLKGKRDILLTR--IGQPLTLEEIE

MGD ψYcf4       QFSSNLAKFLQIPLKEELPFDFFFIFIK
P. minor Ycf4   KQAADLARFLQVGLEG----------IA
```

Supplementary Figure 3. Ycf4 has been pseudogenized in the MGD plastid genome. (A) Ycf4 amino acid sequence is not encoded by a single continuous reading frame. The N-terminus (red) is encoded in +1 reading frame, while the C-terminus (blue) is encoded in +2 reading frame. We found no *ycf4* transcript in the RNA-seq data. (B) Similarity between the Ycf4 amino acid sequences between strain MGD and *P. minor*. The identical amino acid residues between the two sequences are shaded.

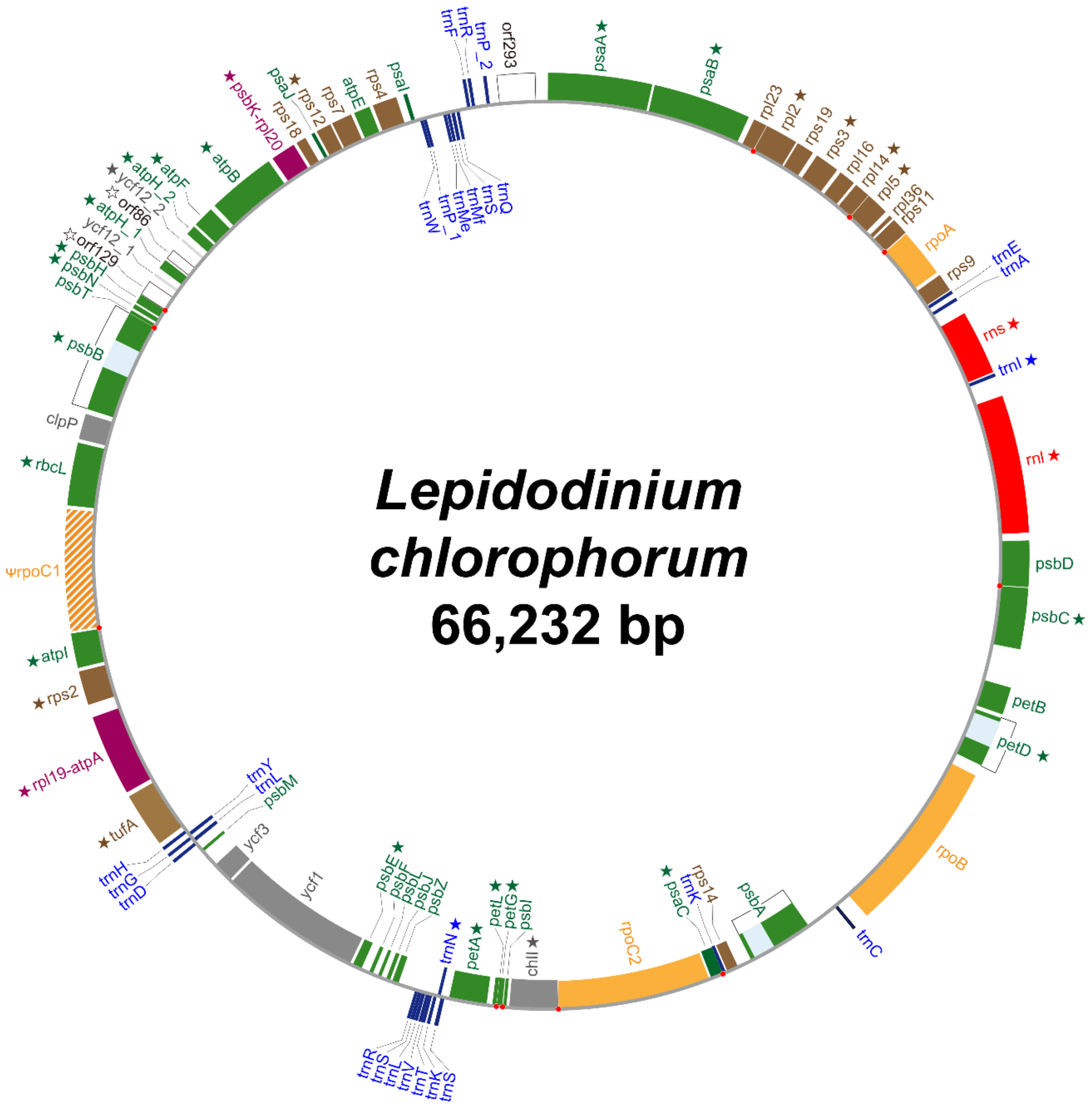

Supplementary Figure 4. Circular map of the plastid genome of *Lepidodinium chlorophorum*. Open reading frames (ORFs) encoding proteins involved in photosynthesis, translation, transcription, and other function are colored in green, brown, orange, and grey, respectively. Functionally unassigned ORFs are shown in white. Fused ORFs are highlighted in purple. rpoC1 is considered as a pseudogene. Ribosomal RNA genes and transfer RNA genes are colored in red and blue, respectively. The ORFs/genes, of which transcripts received RNA editing, are marked by stars. Red dots indicate the overlap of two neighboring ORFs/genes.

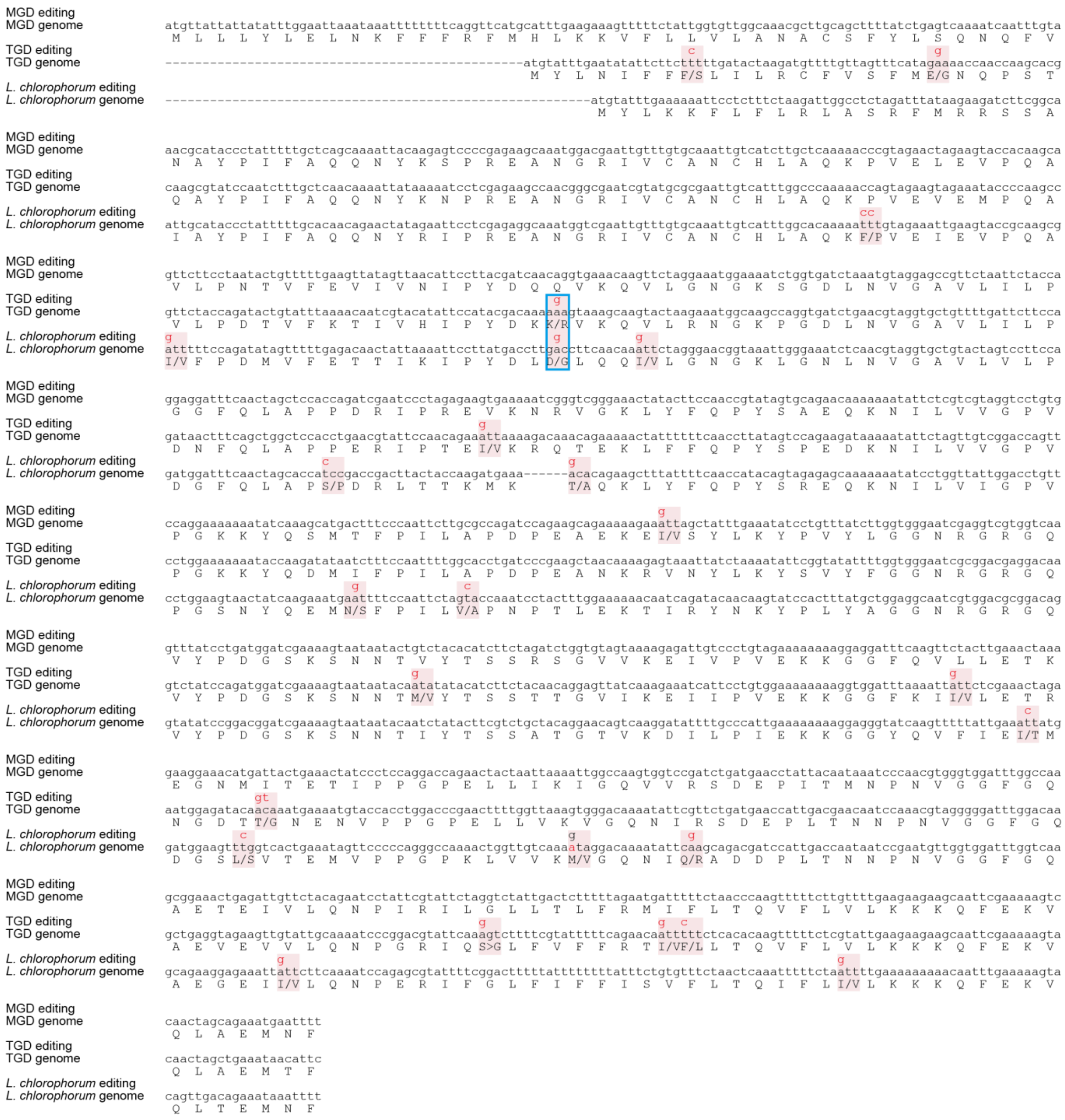

Supplementary Figure 5. One of the positions received base-conversion editing in the *petA* transcripts is shared between strain TGD and *Lepdodinium chlorophorum*. The nucleotides after RNA editing are indicated above the genome sequence. The amino acid changes led by RNA editing are shaded. The editing position shared between strain TGD and *L. chlorophorum* is boxed with a blue line.

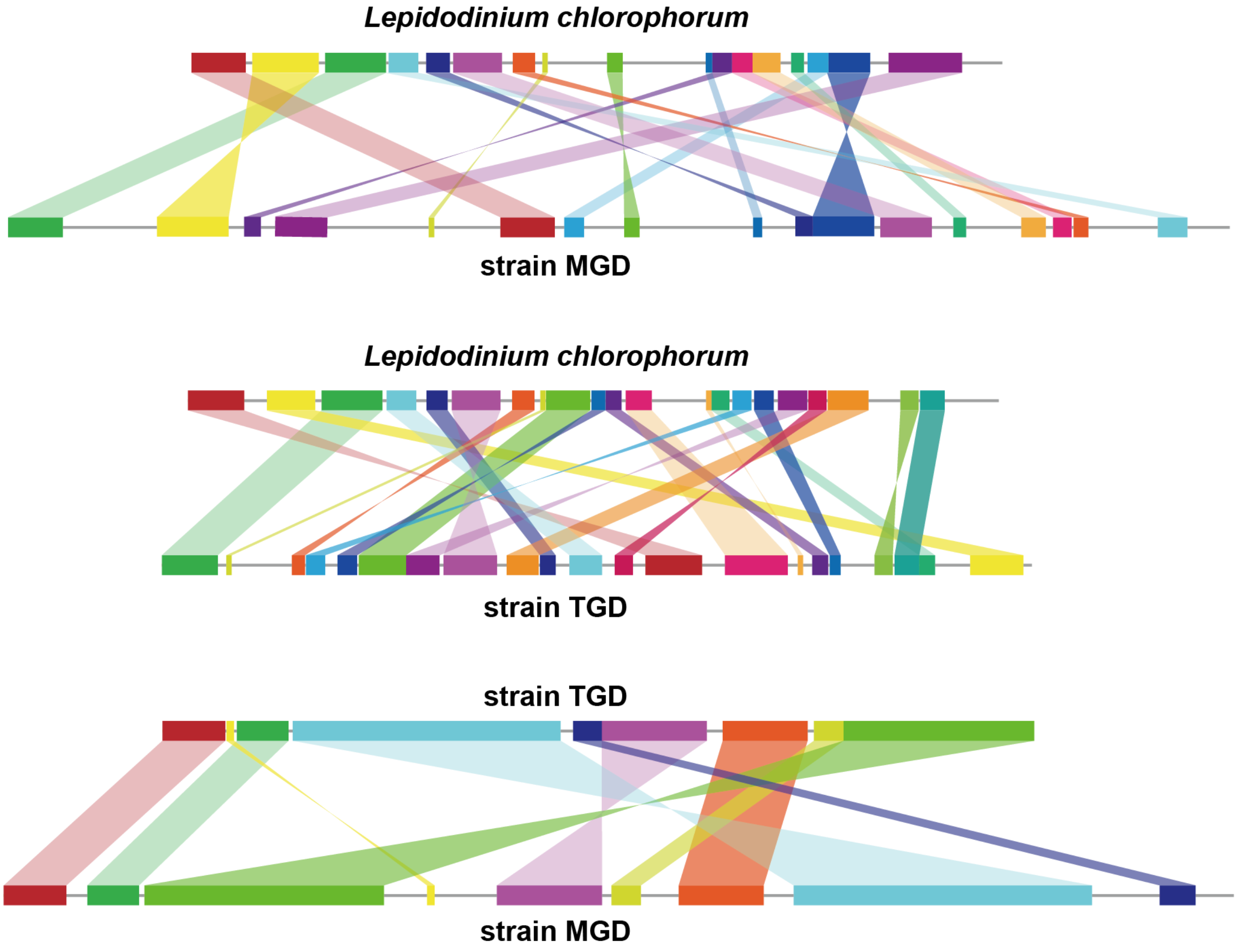

Supplementary Figure 6. Pair-wise comparison of the organization of the peDinoflagellate plastid genomes. Top, synteny between the *Lepdodinium chlorophorum* and MGD plastid genomes. Middle, synteny between the *L. chlorophorum* and TGD plastid genomes. Bottom, synteny between strains TGD and MGD.

Supplementary table 1. Number of the RNA-seq short reads aligned with each of the peDinoflagellate plastid genomes.

	Lepidodinium chlorophorum	**Strain TGD**	**Strain MGD**	***Pedinomonas minor***
Number of the read pair subjected to the mapping ($\times10^6$)	190	202	138	8
Number of the read pair aligned with the plastid genome ($\times10^3$)	95	385	386	373

Supplementary table 2. OFR/gene fusion and overlapping found in the three green peDinoflagellate plastid genomes.

	Lepidodinium chlorophorum	Strain MGD	Strain TGD	*Pedinomonas minor*
Shared at least two genomes	*psbD/psbC*<br>*petL/petG*<br>*rpl14/rpl5*<br>*rpl23/rpl2*	*psbD/psbC*<br>*petL/petG*<br>*rpl14/rpl5−rps8*<br>*rpl23/rpl2*<br>*tufA/rpl19*<br>*psbI/psaI*<br>*trnW/rps18*<br>*rpl5−rps8/rpl36*<br>*rpl36/rpl11*<br>*rpoA−rps9*	*psbD/psbC*<br>*petL−petG*<br><br><br>*tufA/rpl19*<br>*psbI/psaI*<br>*trnW/rps18*<br>*rps8−rpl36*<br>*rps8−rpl36/rps11*<br>*rpoA/rps9*	*psbD/psbC*
Not shared	*rps11/rpoA*<br>*psbB/psbT*<br>*petG/psbI*<br>*chlI/rpoC2*<br>*atpI/rpoC1*<br>*psbH/orf129*<br>*psaC/trnK*<br>*rpl19−atpA*<br>*psbK−rpl20*	*rpl16/rpl14*<br>*psbJ/psbZ*<br>*rpoC1/rpoC2*<br>*rpl5−rps8*<br>*rps19−rps3*<br>*rps12−rps7*	*psbK−psaM/secG*<br>*psbK−psaM/ycf12*<br>*psbL/psbF*<br>*rpl20/rps18*<br>*rpl2/rps19*<br>*rps19/rps3*<br>*psbK−psaM*	*cysA/trnG*

Note: ORF/gene names connected by a dash and a slash indicate ORF/gene fusion and overlapping, respectively.
